# Enhancing computational thinking in early childhood: A cluster-randomized controlled trial of interactive STEM app intervention and the role of engagement

**DOI:** 10.1371/journal.pone.0353980

**Published:** 2026-10-09

**Authors:** Yumei Wang, Shiying Liu

**Affiliations:** 1 School of Marxism, Tangshan Normal University, Tangshan, Hebei, China; 2 School of Economics and Management, North China University of Science and Technology, Tangshan, Hebei, China; UKM: Universiti Kebangsaan Malaysia, MALAYSIA

## Abstract

This cluster-randomized controlled trial investigated whether a 12-week interactive STEM app intervention enhances computational thinking skills among young children aged 4–6 years and whether app usage engagement predicts learning outcomes. Ten classrooms from five kindergartens in Northern China were randomly assigned within each kindergarten to an intervention condition (5 classrooms, 125 children) that received structured app-based activities three times weekly or a waitlist control condition (5 classrooms, 125 children) that continued with traditional educational practices. Computational thinking was assessed at pretest and posttest using the TechCheck-K, a validated assessment designed for young children that does not require prior coding knowledge. App usage engagement was quantified through a multidimensional composite index capturing task completion, time on task, persistence, help-seeking efficiency, navigation independence, self-correction, and attendance. After controlling for baseline ability and demographics, children in the intervention group demonstrated significantly greater computational thinking gains than controls, with a small-to-moderate effect size of Cohen’s *d* = 0.39. Within the intervention group, higher engagement significantly predicted superior posttest performance, although the unique variance explained was modest (approximately 1.5% beyond baseline ability and demographic characteristics) and the relationship is specific to the intervention context. These findings provide evidence that interactive STEM apps can enhance early computational thinking development when implemented systematically, and the main intervention effect was robust to mixed-effects sensitivity analyses accounting for clustering at both the kindergarten and classroom levels. Engagement emerged as a statistically significant, though practically modest, predictor of technology-based learning outcomes within the intervention context, underscoring the potential importance of fostering active, persistent interaction with educational technologies without overstating its standalone contribution. Implications for early childhood education practice, app design, and technology integration are discussed.

## Introduction

Computational thinking—encompassing decomposition, pattern recognition, abstraction, algorithmic design, and debugging—has emerged as a foundational competency for navigating an increasingly digital society [1]. Evidence suggests that foundational computational thinking elements can be cultivated during early childhood, when cognitive architectures are most malleable [2]. However, effective methods for fostering computational thinking in young children remain inadequately understood.

Despite growing recognition of computational thinking’s importance, critical gaps persist in understanding effective intervention approaches for young children. Most existing studies employed small samples (30–50 participants), limiting statistical power and generalizability [1,3]. Intervention durations were typically brief (1–3 weeks), raising questions about whether effects represent genuine learning or task-specific performance [1]. Many investigations lacked control groups, constraining causal inferences [2,3]. Furthermore, most research focused on specialized platforms (ScratchJr, robotics) rather than widely accessible apps that educators could realistically adopt at scale [4].

Technology integration in early childhood education presents unique challenges. Critics caution against excessive screen time, displacement of social interactions and physical play, and question whether young children possess self-regulation skills necessary to benefit from independent technology use [5]. Additionally, abstract computational concepts are inherently challenging for preoperational-stage children, making intervention design complex [6]. These tensions underscore the necessity of rigorous empirical investigations that systematically examine whether digital interventions genuinely enhance young children’s learning outcomes.

Despite these challenges, interactive educational apps offer potentially powerful platforms for developing computational thinking through engaging, developmentally appropriate activities [7]. Well-designed apps provide individualized learning experiences, multimodal representations, low-stakes experimentation, and sustained engagement through game-like mechanics [4]. Research indicates that young children can engage with computational thinking concepts when presented through carefully designed technological scaffolds [1,3]. Contemporary apps’ capacity to automatically log detailed usage data enables systematic examination of engagement patterns and their relationships with learning outcomes—capabilities largely underutilized in developmental research.

Critically, learner engagement—the behavioral, emotional, and cognitive investment children demonstrate during learning activities—serves as a potential predictor of technology-based learning outcomes [8]. Educational technology effectiveness depends not merely on exposure but on how actively and persistently learners engage with materials [7]. Understanding engagement’s role bears substantial practical significance for educational implementation, informing resource allocation, professional development priorities, and intervention design decisions.

This cluster-randomized controlled trial addressed critical gaps by examining whether a structured, 12-week interactive STEM app program enhances computational thinking skills among young children (ages 4–6) compared to traditional practices. Unlike prior brief interventions, we implemented an extended intervention using educational tablet apps across five kindergarten sites, enhancing ecological validity. Critically, we leveraged embedded analytics to examine how children’s multidimensional engagement—incorporating task completion, persistence, time on task, help-seeking, navigation independence, self-correction, and attendance—relates to computational thinking outcomes.

**Research question 1:** What is the effect of a 12-week interactive STEM app intervention on young children’s computational thinking skills compared to traditional educational practices?

**Hypothesis 1:** Children in the intervention group will demonstrate significantly greater improvements in computational thinking skills from pretest to posttest compared to children in the control group.

**Research question 2:** Does the level of app usage engagement predict computational thinking outcomes within the intervention group?

**Hypothesis 2:** Higher levels of app usage engagement will be associated with greater gains in computational thinking skills within the intervention group.

By addressing these questions through a methodologically rigorous design with adequate sample size (*N* = 250), extended duration (12 weeks), validated outcome measures (TechCheck-K), comprehensive covariate controls, and systematic engagement assessment, this study advances understanding of technology-enhanced computational thinking development in early childhood while providing actionable insights for educational practice.

## Method

### Participants

This cluster-randomized pretest-posttest controlled trial was conducted across five kindergartens in Northern China. According to the Ministry of Education of the People’s Republic of China, Northern China contains over 30,000 registered kindergartens serving the early childhood population. The five participating kindergartens were selected through convenience sampling based on established research collaboration agreements between the research team and the kindergarten administrations. Although this purposive approach limits generalizability to the broader population of kindergartens in the region, it ensured the institutional access, administrative support, and implementation fidelity necessary for a rigorous 12-week intervention study. The five sites were located across three municipal districts and varied in size (18–30 children per classroom), socioeconomic catchment area, and curricular emphasis, providing reasonable diversity within the convenience sample. The target population comprised children aged 4–6 years (48–72 months) enrolled during the 2025–2026 academic year. Power analysis for the cluster-randomized design was conducted using the design effect approach to account for within-cluster dependence. The calculation assumed 10 clusters (5 per condition), an average cluster size of 25 children (range: 22–28), a classroom-level intraclass correlation coefficient (ICC) of  .07 (estimated from pilot data), a 1:1 allocation ratio within each kindergarten site, a target effect size of Cohen’s *d* = 0.40, α = .05 (two-tailed), and 15% anticipated attrition. The resulting design effect was 2.68, yielding an effective sample size of approximately 93 participants. Under these assumptions, the estimated power was approximately  .49, which falls below the conventional  .80 threshold. This reflects a recognized limitation of cluster-randomized designs with a small number of clusters: although the total individual-level sample (*N* = 250) would provide  .80 power under a simple randomization design, the effective sample size is substantially reduced by clustering. The achieved sample of 250 children (125 per condition) was the maximum feasible given the participating sites. We note this design-related power constraint as a limitation (see Limitations) and interpret results accordingly, recognizing that the observed statistical significance of the primary intervention effect provides empirical evidence that the design was sufficient to detect the effect in this instance.

Eligibility criteria included: (a) chronological age between 48 and 72 months at the time of pretesting, (b) regular attendance at one of the five participating kindergartens, (c) absence of diagnosed developmental delays or disabilities that would preclude participation in standard classroom activities, and (d) parental consent and child assent. Children were excluded if they had participated in structured computational thinking or coding programs within the six months preceding the study.

This study was approved by the Institutional Review Board of Tangshan Normal University (Protocol No. 2025−81). Written informed consent was obtained from the parents or legal guardians of all participating children, and developmentally appropriate assent was obtained from the children themselves prior to participation.

Fig 1 presents the participant flow, reported in accordance with the CONSORT extension for cluster randomized trials [9]. Across the ten participating classrooms, 268 children were approached; 260 provided parental consent and child assent. Four children were excluded (prior structured computational thinking program participation, *n* = 3; diagnosed developmental delay, *n* = 1), and 256 completed the pretest. Six children were lost before the posttest (family relocation, *n* = 4; prolonged absence, *n* = 2), yielding a final analytic sample of 250 children (intervention: 5 classrooms, 125 children; control: 5 classrooms, 125 children). Randomization of classrooms to conditions was conducted after parental consent was obtained but before baseline assessments commenced; thus, the four excluded children and the six children lost to follow-up were distributed across already-randomized classrooms. Because the six children lost before posttest had no outcome data available, they could not be included in outcome analyses. The primary analysis therefore followed a complete-case approach: all 250 children who completed both pretest and posttest assessments were analyzed in their assigned condition. Among these 250 children, there were no missing pretest or posttest outcome data and no missing demographic questionnaire data; all analyses were conducted on complete cases without imputation. We acknowledge that this approach departs from a strict intention-to-treat framework, and the potential impact of attrition is considered in the Limitations section.

**Fig 1:**
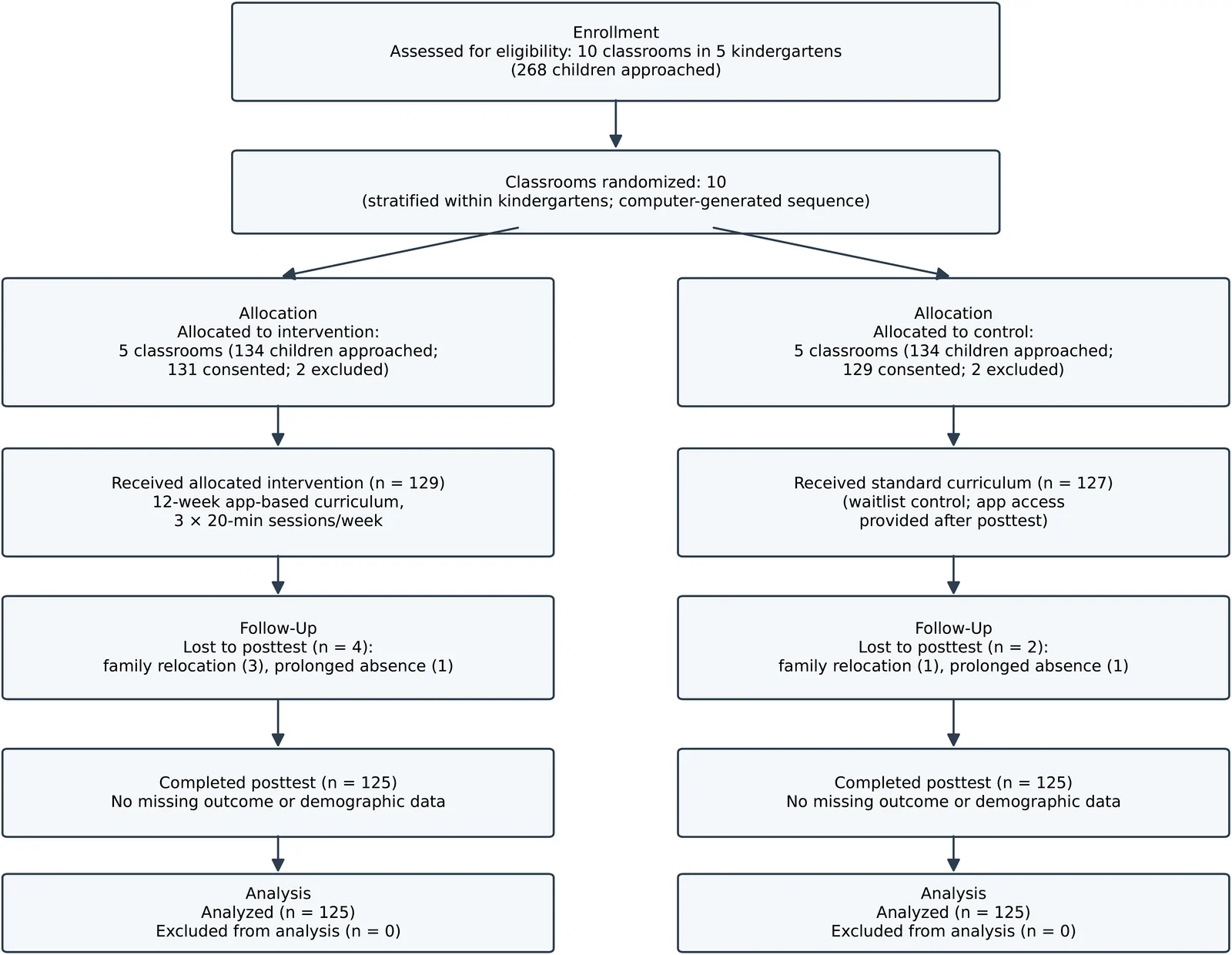
Participant flow diagram. Flow of classrooms and children through enrollment, allocation, follow-up, and analysis, reported in accordance with the CONSORT extension for cluster randomized trials.

This study was approved by the Institutional Review Board of Tangshan Normal University (Protocol No. 2025−81). The following subsection describes the ethical safeguards implemented throughout the study.

### Ethical considerations

#### Informed consent and child assent.

Written informed consent was obtained from the parents or legal guardians of all participating children prior to the commencement of baseline assessments. Consent forms, provided in Mandarin Chinese, described the study’s purpose, procedures, potential risks and benefits, data handling practices, and the right to withdraw at any time without penalty. For child assent, trained research assistants used age-appropriate language accompanied by pictorial aids to explain the study activities to each child individually. Children were told that they would use tablet games during class time and were asked whether they would like to participate. Assent was treated as an ongoing process; children who displayed signs of reluctance or distress during any session—such as verbal refusal, withdrawal from the activity area, or persistent disengagement—were not required to continue. Given the developmental range of the sample (ages 4–6), assent procedures were adapted so that younger children (4-year-olds) received simplified explanations with concrete visual examples, while older children (5- and 6-year-olds) received slightly more detailed descriptions of what participation entailed.

### Digital behavioral data and privacy protection

The multidimensional app engagement data collected through embedded analytics—including task completion, time on task, persistence, help-seeking, navigation independence, self-correction, and session attendance—constituted digital behavioral traces of child users. All behavioral data were automatically anonymized at the point of collection through assignment of randomly generated participant identifiers; no personally identifiable information (names, photographs, or device identifiers) was linked to usage logs. Data were encrypted and stored on password-protected institutional servers with access restricted to authorized members of the research team. These procedures were designed in compliance with the Provisions on the Cyber Protection of Children’s Personal Information (China, 2019), which mandates informed parental consent for the collection of minors’ digital data, purpose limitation, and data minimization principles. Upon completion of the study, raw usage logs were retained in de-identified form for a period consistent with institutional data retention policies and will be securely deleted thereafter.

### Screen time and child wellbeing

The intervention involved three 20-minute sessions per week, totaling approximately 60 minutes of structured screen time weekly. This dosage was designed to fall well within the screen time guidelines issued by the World Health Organization and the American Academy of Pediatrics. For children aged 2–5 years, these organizations recommend limiting sedentary screen time to no more than one hour per day of high-quality programming; for children aged 6 and older, the AAP recommends consistent limits on screen media use. Both organizations emphasize that any screen exposure for young children should be interactive, educational, and co-viewed or facilitated by adults. Each session was embedded within the regular classroom schedule alongside physical activity, social play, and other non-digital learning, ensuring that app use did not displace essential developmental activities. Classroom teachers were instructed to monitor children for signs of visual fatigue, restlessness, or discomfort during sessions. If a child displayed symptoms of fatigue (e.g., eye rubbing, squinting, loss of attention) or expressed a desire to stop, the session was terminated for that child immediately. These incidents were documented on weekly implementation logs to track patterns across the intervention period.

### Adverse event monitoring and withdrawal procedures

Homeroom teachers, who maintained daily contact with participating children, served as the primary monitors for potential adverse events. Teachers were briefed to observe and report any signs of visual strain, behavioral changes (e.g., increased irritability or avoidance of classroom technology), or parental concerns that might be associated with participation in the study. Research staff reviewed adverse event reports during weekly site visits. No child was penalized or disadvantaged for withdrawing or being withdrawn from the study. Participation was voluntary throughout, and families were reminded of their right to discontinue at every assessment point.

### Equity of the control condition

A waitlist control design was employed to address ethical concerns regarding the withholding of potentially beneficial educational resources. During the 12-week intervention period, control group classrooms continued with their standard kindergarten programming, which included typical developmentally appropriate activities. Following the completion of all posttest assessments (Phase 3), control group classrooms received full access to the intervention apps and structured implementation materials for a four-week period (Phase 4, Weeks 13–16). Although this delayed access period was shorter than the 12-week intervention, it was designed to provide all participating children with the core educational experience while preserving the internal validity of the study. Teachers in the control group were offered the same training session and implementation guide as intervention group teachers. This approach balanced the scientific requirement for an uncontaminated control condition with the ethical imperative that all participants have the opportunity to benefit from the educational resources under investigation.

### Design and procedure

#### Research design.

This study employed a cluster-randomized pretest-posttest controlled trial. The five participating kindergartens were recruited through convenience sampling (see Participants), and the classroom served as the unit of randomization. Two classrooms were enrolled in each kindergarten, yielding ten classrooms in total (mean = 25 children per classroom; range = 22–28). Within each kindergarten, the two classrooms were randomly assigned, one to the intervention condition and one to the control condition. Because participating sites were not randomly sampled from the broader population of kindergartens, the design is best characterized as a cluster-randomized controlled trial with convenience sampling of participating sites [9]. The independent variable was intervention condition (interactive STEM app intervention vs. traditional educational practice), and the dependent variable was computational thinking skill as measured by TechCheck-K. App usage engagement served as a continuous predictor variable within the intervention group, measured throughout the 12-week intervention period.

### Assignment and Group Allocation

Within each of the five participating kindergartens, the two participating classrooms were matched on baseline characteristics (class size, teacher experience, baseline STEM curriculum emphasis), and one classroom was then randomly assigned to the intervention condition and the other to the control condition using a computer-generated random number sequence. This stratified (within-site) assignment procedure yielded five intervention classrooms (125 children) and five control classrooms (125 children), ensured representation of both conditions within each site, and controlled for potential site-level confounds. Allocation concealment was maintained until baseline assessments were completed.

The intervention group received a structured 12-week interactive STEM app program integrated into their regular classroom activities, while the control group continued with their kindergarten’s existing educational practices without access to the intervention apps. Control group teachers were instructed to maintain their typical instructional approaches and were offered access to the intervention materials following the completion of posttest assessments (waitlist control).

### Timeline and procedures

#### Data collection occurred across four phases over a 14-week period.

**Phase 1: Baseline Assessment (Week 0).** Trained research assistants administered the TechCheck-K assessment individually to all participating children in a quiet area of their kindergarten. Each assessment session lasted approximately 20 minutes. Parents completed a demographic questionnaire providing information on child age, gender, parental education (father and mother), household income, and primary home language.

**Phase 2: Intervention Implementation (Weeks 1–12).** Children in the intervention group engaged with interactive STEM apps during designated activity periods three times per week, with each session lasting approximately 20 minutes (total planned dosage: 720 minutes over 12 weeks). The intervention was delivered on tablet devices provided by the research team and installed with three educational apps selected for their alignment with computational thinking competencies: a sequencing and algorithmic thinking app, a pattern recognition and mathematical reasoning app, and a debugging and problem-solving app. All three apps ran on Android tablets with a Mandarin Chinese interface, icon-based navigation, and audio prompts, minimizing literacy demands for pre-literate children. Apps were selected and configured according to the following criteria: (a) developmental appropriateness for children aged 4–6 years; (b) explicit alignment with the targeted computational thinking competencies; (c) immediate, multimodal feedback and progressive difficulty structures; (d) minimal text and symbolic abstraction; (e) absence of advertisements, in-app purchases, and external links; and (f) offline functionality to ensure standardized implementation across sessions. Table 1 summarizes each app’s design focus, targeted computational thinking competencies, core mechanics, representative activities, and curriculum phase emphasis. Detailed activity protocols and technical specifications of the apps are available from the corresponding author upon request.

**Table 1:** Characteristics of the three educational apps used in the intervention.

App	Design Focus	Targeted CT Competencies	Core Mechanics	Representative Activities	Curriculum Phase Emphasis
App 1	Sequencing and algorithmic thinking	Algorithms; control structures	Drag-and-drop ordering of action blocks with immediate visual execution feedback	Arrange instruction blocks to guide a character through a route; order everyday routines (e.g., steps of brushing teeth) into correct sequences	Foundation (Weeks 1–3); continued practice in Application (Weeks 4–6)
App 2	Pattern recognition and mathematical reasoning	Pattern recognition; abstraction; representation	Identifying, completing, and extending visual and numeric patterns; rule-based sorting and classification	Complete ABAB and AABB patterns; classify objects by multiple attributes; transfer pattern rules to novel stimuli	Application (Weeks 4–6)
App 3	Debugging and problem-solving	Debugging; decomposition; evaluation	Locating and correcting errors in pre-set sequences; comparing alternative solution paths	Identify the incorrect step in a sequence and repair it; decompose multi-step problems into components; evaluate and justify the better of two solution paths	Analysis (Weeks 7–9); integrated use in Creation (Weeks 10–12)

*Note.* CT = computational thinking. All apps were installed on Android tablet devices provided by the research team and used a Mandarin Chinese interface with icon-based navigation and audio prompts. Detailed activity protocols and technical specifications are available from the corresponding author upon request.

### Intervention curriculum design

The 12-week intervention curriculum was designed according to Bloom’s Revised Taxonomy of cognitive processes [10], adapted for early childhood developmental characteristics, to ensure a systematic progression from lower-order to higher-order computational thinking competencies. Four instructional phases were sequenced to scaffold children’s cognitive engagement from foundational recognition tasks toward creative application of computational concepts. Table 2 presents the alignment between intervention phases, Bloom’s cognitive levels, targeted computational thinking competencies, representative app activities, and teacher facilitation strategies.

**Table 2:** Intervention curriculum framework aligned with bloom’s revised taxonomy.

Phase	Weeks	Bloom’s Cognitive Level	CT Competency Targeted	Representative App Activities	Teacher Facilitation Strategies
Foundation	1–3	Remember, Understand	Identify sequences; recognize repeating patterns; recall basic interface operations	Follow demonstrated sequences to complete sorting tasks; match and identify repeating visual patterns; navigate basic app menus	Model app operations step-by-step; use directed questioning (“What comes next?”); provide visual demonstrations of task goals
Application	4–6	Apply	Execute simple algorithms independently; apply learned patterns to novel contexts	Complete multi-step sequencing tasks without demonstration; transfer pattern rules to unfamiliar stimuli; solve simple pathway problems	Pose open-ended questions (“How would you do this?”); gradually reduce scaffolding; encourage independent attempts before assistance
Analysis	7–9	Analyze	Decompose complex tasks; identify and correct errors (debugging); compare alternative solution paths	Locate errors in pre-set sequences and correct them; break multi-step problems into components; evaluate why one solution path succeeds and another fails	Prompt verbalization of reasoning (“Where did it go wrong? Why?”); encourage error identification before correction; facilitate peer discussion of strategies
Creation	10–12	Evaluate, Create	Design original sequences; evaluate solution quality; synthesize multiple CT competencies	Create novel patterns and sequences; evaluate peer-designed challenges; combine sequencing, pattern recognition, and debugging in integrated tasks	Support collaborative peer activities; facilitate reflective discussions (“Which solution works better? Why?”); encourage children to design challenges for peers

The three educational apps were used across all four phases; however, the emphasis shifted across the intervention period. During the Foundation phase (Weeks 1–3), activities focused primarily on the sequencing app to establish basic procedural understanding. The Application phase (Weeks 4–6) introduced increasing use of the pattern recognition app alongside continued sequencing practice. The Analysis phase (Weeks 7–9) emphasized the debugging app while integrating skills acquired in prior phases. The Creation phase (Weeks 10–12) required children to draw upon all three apps in integrated tasks that demanded synthesis of multiple computational thinking competencies. This progressive structure ensured that earlier skills served as building blocks for more complex cognitive operations, consistent with the hierarchical nature of Bloom’s taxonomy and developmental learning trajectory frameworks in early childhood education [11,12].

Classroom teachers received a two-hour training session prior to intervention implementation, covering app functionality, facilitation strategies (e.g., open-ended questioning, scaffolding), and technical troubleshooting. Teachers were provided with a structured implementation guide specifying weekly learning objectives and suggested facilitation prompts. Research staff conducted weekly site visits to monitor implementation fidelity, address technical issues, and provide ongoing support.

Control group participants continued with their kindergarten’s standard educational programming, which included typical developmentally appropriate activities but did not involve structured computational thinking instruction or the specific intervention apps.

**Phase 3: Posttest Assessment (Week 12).** At the conclusion of the 12-week intervention period, all participating children completed the TechCheck-K posttest assessment following the same protocol as the pretest. Assessors were blind to participants’ group assignment.

**Phase 4: Waitlist Control Access (Weeks 13–16).** Following posttest data collection, control group classrooms were provided access to the intervention apps and materials for a four-week period as a delayed treatment condition, ensuring ethical equipoise.

### Implementation fidelity

To ensure intervention integrity, implementation fidelity was monitored through multiple methods: (a) automated tracking of session frequency and duration via app analytics, (b) teacher completion of weekly implementation logs documenting planned versus actual sessions and any deviations from protocol, and (c) direct observation of 20% of intervention sessions by trained research staff using a standardized checklist. Fidelity criteria specified that at least 80% of planned sessions should be completed, with children having functional access to devices and teachers providing appropriate facilitation.

### Bias control

Several procedures were implemented to minimize threats to internal validity. First, selection bias was addressed through stratified random assignment of classrooms within each kindergarten site, matching on class size, teacher experience, and baseline STEM curriculum emphasis prior to randomization. Baseline equivalence was verified through independent samples *t* tests and chi-square analyses on all demographic and pretest variables. Second, assessment bias was controlled by ensuring that all posttest assessors were blind to participants’ group assignment; assessors were not informed which classrooms had received the intervention. Third, to minimize experimenter expectancy effects, classroom teachers in the intervention group were trained on app functionality and general facilitation strategies but were not informed of the specific study hypotheses regarding engagement or expected effect sizes. Fourth, potential Hawthorne effects were mitigated by maintaining a consistent research presence across both conditions; research staff conducted site visits to all classrooms (intervention and control) on the same schedule. Fifth, testing effects were controlled by using identical assessment protocols and assessors at pretest and posttest, with a 12-week interval between administrations to reduce practice effects. Sixth, contamination between conditions was minimized through classroom-level (rather than individual-level) assignment and by restricting the intervention apps to devices that were collected at the end of each session, preventing informal sharing across classrooms. Finally, statistical controls for potential confounders included baseline computational thinking scores, child age, gender, parental education, household income, home language, and kindergarten site in all regression models.

### Measures

#### Computational thinking: TechCheck-K.

The primary outcome measure was TechCheck-K [13], a validated unplugged assessment of computational thinking skills designed for young children. The instrument was translated into Mandarin Chinese for use in the present study following standard forward-backward translation procedures. TechCheck-K is a modified version of the original TechCheck instrument, adapted for younger children by reducing the number of response alternatives from four to three per item and using exclusively non-verbal pictorial response options to accommodate pre-literate children. The assessment consists of 15 multiple-choice items drawn from six computational thinking domains: algorithms, modularity, control structures, representation, hardware/software, and debugging. Each correct response is awarded one point with no penalty for incorrect answers, yielding a total raw score ranging from 0 to 15. In the present study, raw scores were converted to a percentage-correct scale (0–100) to facilitate interpretation. The assessment is administered individually by a trained assessor and requires approximately 20 minutes to complete.

Relkin et al. (2020) reported strong psychometric properties for the original TechCheck instrument [14], including internal consistency reliability (Cronbach’s α = .85), test-retest reliability over a two-week interval (*r* = .82), and construct validity evidenced by significant correlations with age (*r* = .58) and teacher-rated problem-solving skills (*r* = .61); the kindergarten version used in the present study has subsequently been validated and normed [13]. The measure has demonstrated sensitivity to instructional interventions in early childhood settings and has been used across multiple countries in educational and research contexts.

In the present study, all assessors completed standardized training including practice administrations with feedback. The assessment was administered using tablet devices with a touchscreen interface, allowing children to select responses by tapping their chosen option. Inter-rater reliability was established by having 20% of assessments independently double-scored from video recordings. Intraclass correlation coefficients (ICC) for total scores exceeded  .85, indicating strong inter-rater agreement.

### Demographic and control variables

Parents completed a questionnaire assessing child and family demographic characteristics used as control variables in analyses: Child age was calculated as the number of months between date of birth and pretest date. Parental education was assessed separately for fathers and mothers using a 6-point ordinal scale: 1 = *less than high school*, 2 = *high school diploma*, 3 = *some college*, 4 = *bachelor’s degree*, 5 = *master’s degree*, 6 = *doctoral or professional degree*. Household income was measured on a 6-point ordinal scale reflecting total annual household income before taxes (in Chinese Yuan): 1 = *less than ¥25,000*, 2 = *¥25,000–¥49,999*, 3 = *¥50,000–¥74,999*, 4 = *¥75,000–¥99,999*, 5 = *¥100,000–¥149,999*, 6 = *¥150,000 or more*. Primary home language was recorded as a binary variable (1 = Mandarin Chinese, 0 = other), reflecting whether the primary language spoken at home was the language of instruction used in the participating kindergartens.

### App engagement index

App usage engagement was operationalized as a multidimensional composite index derived from behavioral data automatically logged by the three intervention apps throughout the 12-week implementation period. Seven component indicators were recorded for each child in the intervention group: (a) task completion rate (percentage of assigned activities completed per session, 0–100%); (b) time on task (mean active engagement time per session in minutes, range: 10–25); (c) persistence (mean number of retry attempts following incorrect responses before requesting help, range: 1–5); (d) help-seeking efficiency (percentage of help requests that were contextually appropriate rather than reflexive, 0–100%); (e) navigation independence (proportion of app transitions completed without adult assistance, 0–100%); (f) self-correction rate (percentage of errors self-corrected before submission, 0–100%); and (g) attendance (percentage of scheduled sessions attended, 0–100%).

Each component was aggregated across the 12-week period to yield a single per-child value, then normalized to a 0–100 scale using min-max transformation within the intervention sample. The seven normalized components were combined using equal weights (i.e., the unweighted arithmetic mean) to produce a composite engagement index ranging from 0 to 100. Equal weighting was adopted because no prior empirical or theoretical basis existed for differential weighting of these components in early childhood app-based learning contexts. Internal consistency of the seven-component index was strong (Cronbach’s α = .90, standardized α = .96), and inter-item correlations ranged from  .68 to  .88, supporting the coherence of the composite. All 125 intervention-group children had complete data on all seven components; no imputation was required at the component level. For analysis purposes, the composite index was mean-centered prior to entry into regression models.

### Data analysis

All statistical analyses were conducted using R Version 4.1.1 with significance level set at α = .05 (two-tailed). Prior to hypothesis testing, preliminary analyses examined data quality, distributional properties, and assumptions underlying primary statistical models.

To test the hypothesis that children in the intervention group would demonstrate significantly greater improvements in computational thinking skills than controls (H1), a multiple regression analysis was conducted with posttest CT score as the dependent variable. The model included intervention group (0 = control, 1 = intervention) as the primary predictor, pretest CT score as a covariate to control for baseline differences and increase statistical power, and demographic control variables (age, gender, father’s education, mother’s education, household income, home language, kindergarten site entered as dummy-coded variables).

### Assumptions and diagnostics

For all regression models, assumptions of linearity, independence of errors, homoscedasticity, normality of residuals, and absence of multicollinearity were assessed through examination of residual plots, variance inflation factors (VIF < 10), Durbin-Watson statistics, and normal probability plots. Influential cases were identified using Cook’s distance (*D* > 4/*n*) and leverage values, with sensitivity analyses conducted excluding outliers to determine their impact on parameter estimates.

Given the nested structure of the data (children within classrooms within kindergartens), intraclass correlation coefficients (ICCs) were calculated from unconditional variance-component models and from models adjusted for condition and covariates to quantify the proportion of variance in CT scores attributable to clustering at the kindergarten and classroom levels. Three considerations informed the use of conventional regression with kindergarten site entered as fixed-effect covariates as the primary analytic approach. First, the number of higher-level units was small (five kindergartens; ten classrooms), and random-effects variance estimates are known to be biased and imprecise with so few clusters; fixed-effects approaches are recommended under such conditions [15]. Second, because classrooms were randomly assigned within each kindergarten, including site as dummy-coded covariates directly mirrors the randomized-blocks structure of the design and absorbs site-level heterogeneity. Third, to verify that conclusions did not depend on this analytic choice, all primary models were re-estimated as linear mixed-effects models. Two sensitivity specifications were examined: (a) random intercepts for kindergarten site only, and (b) random intercepts for classroom nested within kindergarten site, the latter directly corresponding to the unit of randomization. In addition, a cluster-level analysis was conducted in which covariate-adjusted classroom means were compared using a paired *t* test within each kindergarten site (see Results).

## Results

### Descriptive statistics and group comparisons

Descriptive statistics for all study variables are presented in Table 3. As shown in Fig 1, of the 268 children approached across the ten participating classrooms, 260 were consented, 4 were excluded, and 6 were lost before the posttest. The final analytic sample comprised 250 children (control group: 5 classrooms, *n* = 125; intervention group: 5 classrooms, *n* = 125) aged 48–72 months (*M* = 66.50 months, *SD* = 10.47), with 22–28 children per classroom. The sample was approximately gender-balanced, with 113 girls (45.2%) and 137 boys (54.8%).

**Table 3:** Descriptive statistics and group comparisons at baseline.

Variable	Control Group (*n* = 125)	Intervention Group (*n* = 125)	Test Statistic	*p*	Effect Size
Age in months, *M* (*SD*)	65.45 (10.67)	67.54 (10.18)	*t*(247) = −1.58	.115	*d* = 0.20
Gender, *n* (%)			χ²(1) = 0.26	.611	
Female	59 (47.2%)	54 (43.2%)			
Male	66 (52.8%)	71 (56.8%)			
Father’s education, *n* (%)			χ²(5) = 1.66	.894	
Less than high school	9 (7.2%)	9 (7.2%)			
High school diploma	11 (8.8%)	16 (12.8%)			
Some college	23 (18.4%)	26 (20.8%)			
Bachelor’s degree	43 (34.4%)	41 (32.8%)			
Master’s degree	27 (21.6%)	23 (18.4%)			
Doctoral/professional	12 (9.6%)	10 (8.0%)			
Mother’s education, *n* (%)			χ²(5) = 1.84	.871	
Less than high school	5 (4.0%)	7 (5.6%)			
High school diploma	12 (9.6%)	10 (8.0%)			
Some college	22 (17.6%)	28 (22.4%)			
Bachelor’s degree	50 (40.0%)	44 (35.2%)			
Master’s degree	20 (16.0%)	18 (14.4%)			
Doctoral/professional	16 (12.8%)	18 (14.4%)			
Household income, *n* (%)			χ²(5) = 9.25	.099	
<¥25,000	18 (14.4%)	9 (7.2%)			
¥25,000–49,999	19 (15.2%)	23 (18.4%)			
¥50,000–74,999	30 (24.0%)	31 (24.8%)			
¥75,000–99,999	23 (18.4%)	38 (30.4%)			
¥100,000–149,999	22 (17.6%)	14 (11.2%)			
≥ ¥150,000	13 (10.4%)	10 (8.0%)			
Home language: Mandarin Chinese, *n* (%)	112 (89.6%)	114 (91.2%)	χ²(1) = 0.19	.662	
Pretest CT score, *M* (*SD*)	52.35 (10.18)	53.21 (11.05)	*t*(246) = −0.64	.525	*d* = 0.08
Posttest CT score, *M* (*SD*)	60.71 (11.02)	65.26 (12.02)	*t*(246) = −3.12	.002	*d* = 0.39
App engagement index, *M* (*SD*)	—	72.45 (12.38)	—	—	—

*Note.* CT = computational thinking. CT scores are reported on a percentage-correct scale (0–100) derived from the 15-item TechCheck-K. Household income reflects total annual household income before taxes in Chinese Yuan. App engagement index available only for intervention group participants. Effect sizes: *d* = Cohen’s *d*, *V* = Cramér’s *V*. Chi-square tests were used for categorical variables; independent samples *t* tests were used for continuous variables.

Baseline equivalence tests revealed no significant differences between groups on demographic or pretest variables. Independent samples *t* tests indicated that groups did not differ significantly on age (*t*(247) = −1.58, *p* = .115, *d* = 0.20) or pretest computational thinking scores (*t*(246) = −0.64, *p* = .525, *d* = 0.08). Chi-square analyses confirmed no significant group differences in gender distribution (χ²(1) = 0.26, *p* = .611), father’s education (χ²(5) = 1.66, *p* = .894), mother’s education (χ²(5) = 1.84, *p* = .871), household income distribution (χ²(5) = 9.25, *p* = .099), or home language (χ²(1) = 0.19, *p* = .662). These findings support the assumption that the assignment procedure successfully created equivalent groups at baseline.

Both groups demonstrated improvements in computational thinking from pretest to posttest; however, the magnitude of improvement differed between conditions. The control group improved from a pretest mean of 52.35 (*SD* = 10.18) to a posttest mean of 60.71 (*SD* = 11.02), representing a mean gain of 8.36 points. The intervention group improved from a pretest mean of 53.21 (*SD* = 11.05) to a posttest mean of 65.26 (*SD* = 12.02), representing a mean gain of 12.05 points. The difference in posttest scores between groups was statistically significant (*t*(246) = −3.12, *p* = .002, *d* = 0.39), with the intervention group scoring approximately 4.55 points higher than the control group at posttest. The improvement observed in both groups likely reflects a combination of natural developmental maturation, repeated testing, and ongoing kindergarten instruction, while the differential improvement in the intervention group is attributable to the app-based intervention.

### Bivariate correlations

Pearson correlation coefficients among primary study variables are displayed in Table 4. As expected, pretest and posttest computational thinking scores were strongly positively correlated (*r* = .80, *p* < .001), indicating substantial stability in children’s relative standing over the 12-week period. Posttest CT scores demonstrated small-to-moderate positive correlations with age (*r* = .27, *p* < .001), father’s education (*r* = .29, *p* < .001), mother’s education (*r* = .17, *p* < .01), and household income (*r* = .20, *p* < .01), supporting the inclusion of these variables as covariates.

**Table 4:** Correlations, means, and standard deviations for study variables.

Variable	*M*	*SD*	1	2	3	4	5	6	7	8
1. Pretest CT	52.78	10.62	—							
2. Posttest CT	62.99	11.61	.80***	—						
3. Age	66.50	10.47	.24***	.27***	—					
4. Gender	0.55	0.50	−.01	.00	.09	—				
5. Father’s education	3.55	1.38	.19**	.29***	−.10	−.11	—			
6. Mother’s education	3.62	1.33	.11	.17**	−.04	.01	−.02	—		
7. Household income	3.37	1.45	.20**	.20**	−.07	.03	.09	.02	—	
8. App engagementa	72.45	12.38	.40***	.47***	.20*	.11	.09	.05	.05	—

*Note. N* = 250 for Variables 1–7. CT = computational thinking. Gender coded as 0 = *female*, 1 = *male*. Home language omitted from table for parsimony (see Table 3 for group comparison). aApp engagement index available only for intervention group (*n* = 125).*p < .05. **p < .01. ***p < .001 (two-tailed).

Within the intervention group, app usage engagement was significantly correlated with posttest CT scores (*r* = .47, *p* < .001), pretest CT scores (*r* = .40, *p* < .001), and age (*r* = .20, *p* < .05), suggesting that higher-performing and older children tended to exhibit greater engagement with the interactive STEM apps. Variance inflation factor (VIF) values for all predictors in the primary regression model ranged from 1.01 to 1.21, well below the threshold of 10, indicating that multicollinearity was not a concern.

### Primary analyses

#### Main intervention effect.

To address the first research question examining whether the interactive STEM app intervention enhanced computational thinking skills compared to traditional educational practices, a multiple regression analysis was conducted with posttest CT score as the dependent variable. The model included intervention group as the primary predictor, pretest CT score as a covariate, and demographic variables (age, gender, parental education, household income, home language) and kindergarten site (dummy-coded) as additional covariates.

Results of the regression analysis are presented in Table 5. After controlling for pretest CT scores and all demographic covariates, intervention group membership was a significant positive predictor of posttest CT scores (*B* = 3.75, *SE* = 0.72, *t* = 5.17, *p* < .001, 95% CI [2.32, 5.18]). This coefficient indicates that, on average, children in the intervention group scored 3.75 points higher on the posttest CT assessment than children in the control group, holding all other variables constant. The unadjusted between-group effect size was Cohen’s *d* = 0.39 (computed from posttest group means and pooled standard deviation), and the intervention group variable accounted for a substantial portion of variance in posttest scores (partial η² = .10).

**Table 5:** Multiple regression predicting posttest computational thinking scores (RQ1).

Predictor	*B*	*SE*	95% CI	*t*	*p*
Intercept	34.03	3.65	[26.84, 41.21]	9.33	< .001
Pretest CT (centered)	0.76	0.04	[0.68, 0.83]	20.31	< .001
Group (intervention)	3.75	0.72	[2.32, 5.18]	5.17	< .001
Age	0.15	0.04	[0.08, 0.22]	4.05	< .001
Gender (male)	0.88	0.74	[−0.58, 2.33]	1.19	.237
Father’s education	1.51	0.28	[0.95, 2.06]	5.34	< .001
Mother’s education	1.08	0.28	[0.53, 1.64]	3.84	< .001
Household income	0.39	0.26	[−0.12, 0.90]	1.50	.136
Home language (Chinese)	4.23	1.25	[1.76, 6.69]	3.38	< .001

*Note. N* = 250. *R*² = .777, Adjusted *R*² = .766, *F*(12, 237) = 68.78, *p* < .001. CT = computational thinking. Site dummy variables (Site 2–5) included as covariates but not displayed for parsimony. Pretest CT score was mean-centered. Cohen’s *d* for unadjusted between-group posttest comparison = 0.39.

The overall regression model was statistically significant, *F*(12, 237) = 68.78, *p* < .001, and explained 77.7% of the variance in posttest CT scores (*R*² = .777, adjusted *R*² = .766). As expected, pretest CT score was the strongest predictor (*B* = 0.76, *t* = 20.31, *p* < .001), indicating substantial continuity in computational thinking skills over the intervention period. Among demographic covariates, age (*B* = 0.15, *p* < .001), father’s education (*B* = 1.51, *p* < .001), mother’s education (*B* = 1.08, *p* < .001), and home language (*B* = 4.23, *p* < .001) emerged as significant predictors, whereas gender and household income did not significantly predict posttest scores after controlling for other variables.

These findings provide strong support for Hypothesis 1, demonstrating that children who participated in the 12-week interactive STEM app intervention exhibited significantly greater improvements in computational thinking skills compared to peers receiving traditional instruction.

### Engagement effect within intervention group

The second research question examined whether level of app usage engagement predicted computational thinking gains within the intervention group. A multiple regression analysis restricted to intervention group participants (*n* = 125) was conducted with posttest CT score as the dependent variable and centered pretest CT score, centered app engagement index, and demographic covariates as predictors.

Results presented in Table 6 revealed that app usage engagement was a significant positive predictor of posttest CT scores after controlling for pretest scores and covariates (*B* = 0.14, *SE* = 0.05, *t* = 2.91, *p* = .004, 95% CI [0.04, 0.23]). This coefficient indicates that for every 1-point increase in the app engagement index (on the 0–100 scale), children’s posttest CT scores increased by an average of 0.14 points, holding other variables constant.

**Table 6:** Hierarchical regression predicting posttest CT scores: Engagement effect within intervention group (RQ2).

Predictor	*B*	*SE*	β	*B*	*SE*	β
Intercept	10.84	4.52	—	9.28	4.38	—
Pretest CT (centered)	0.76	0.05	.67	0.73	0.05	.64
App engagement (centered)	—	—	—	0.14	0.05	.17
Age	0.18	0.09	.18	0.15	0.09	.15
Gender (male)	0.89	1.52	.04	0.95	1.47	.04
Father’s education	0.64	0.51	.08	0.58	0.49	.07
Mother’s education	0.38	0.52	.05	0.29	0.50	.03
Household income	0.27	0.47	.03	0.22	0.45	.03
Home language (Chinese)	1.86	1.54	.08	1.72	1.49	.07
Model Statistics						
*R*²	.780			.796		
Adjusted *R*²	.759			.774		
Δ*R*²	—			.018**		
*F* for Δ*R*²	—			8.44**		

*Note. n* = 125 (intervention group only). CT = computational thinking. Pretest CT and app engagement index were mean-centered. Site dummy variables (Site 2–5) included as covariates but not displayed. β = standardized regression coefficient. Cohen’s *f*² = 0.075 (small-to-medium effect size). Model 1 df: *F*(11, 113); Model 2 df: *F*(12, 112). Δ*R*² represents the change in variance explained when app engagement is added to Model 2.**p < .01.

A hierarchical regression approach further clarified the unique contribution of engagement. Model 1, which included only pretest CT and demographic covariates (with site dummy variables), explained 78.0% of variance in posttest scores (*R*² = .780). Adding the app engagement index in Model 2 significantly improved model fit, Δ*R*² = .015, *F*(1, 112) = 8.44, *p* = .004, Cohen’s *f*² = 0.075 (small-to-medium effect). This indicates that app engagement accounted for approximately 1.5% of unique variance in posttest CT scores beyond what was explained by baseline ability and demographics.

These findings support Hypothesis 2, demonstrating that children who exhibited higher levels of engagement with the interactive STEM apps—characterized by greater task completion rates, time on task, persistence, self-correction, and attendance—achieved significantly stronger computational thinking outcomes. Importantly, because engagement could only be measured among children who participated in the app-based activities, this predictive relationship is specific to the context of the present intervention and should not be interpreted as a universal or causal association between engagement and computational thinking applicable to all young learners.

### Exploratory gender subgroup analysis

To examine whether intervention effects differed by gender, an exploratory analysis was conducted by adding a gender × intervention group interaction term to the primary regression model. The interaction was not statistically significant (*B* = 1.52, *SE* = 1.48, *t* = 1.03, *p* = .305), indicating that the intervention effect on computational thinking did not differ significantly between boys and girls. Subgroup analyses conducted separately by gender revealed that the direction of the intervention effect was positive for both groups: among girls (*n* = 113), the intervention coefficient was *B* = 2.85 (*p* = .014), and among boys (*n* = 137), the intervention coefficient was *B* = 4.48 (*p* < .001). Within the intervention group, app engagement significantly predicted posttest computational thinking scores for girls (*B* = 0.26, *p* = .003, *n* = 54), though not for boys (*B* = 0.07, *p* = .332, *n* = 71). These exploratory findings suggest that the intervention benefited boys and girls comparably, though future research with larger samples would be needed to detect potential small gender-differentiated effects.

Taken together, results provided strong support for both primary hypotheses. Children who participated in the 12-week interactive STEM app intervention demonstrated significantly greater improvements in computational thinking skills compared to peers in the control group, with a small-to-moderate effect size (Cohen’s *d* = 0.39). Within the intervention group, higher levels of app usage engagement were associated with superior posttest performance, accounting for approximately 1.5% of unique variance beyond baseline ability and demographics.

### Clustering diagnostics and sensitivity analyses

To evaluate the appropriateness of the primary ordinary least squares (OLS) regression approach given the hierarchical data structure, intraclass correlation coefficients (ICCs) were computed at the kindergarten and classroom levels using variance-component models. At baseline, clustering was negligible at both levels (kindergarten ICC < .01; classroom ICC < .01). At posttest, unadjusted ICCs were  .04 at the kindergarten level and  .12 at the classroom level; the classroom-level coefficient partly reflects between-classroom differences attributable to condition itself, because condition was assigned at the classroom level. After adjusting for condition and covariates in a three-level model (children within classrooms within kindergartens), ICCs were  .19 at the kindergarten level and  .09 at the classroom level.

Because the number of clusters was small (five kindergartens, ten classrooms), primary analyses relied on OLS regression with kindergarten site entered as dummy-coded fixed effects, as recommended for designs with few clusters [15]. To verify that conclusions did not depend on this choice and to explicitly address the dependence induced by classroom-level randomization, three sensitivity analyses were conducted: (a) linear mixed-effects models with random intercepts for kindergarten site, (b) linear mixed-effects models with random intercepts for classroom nested within kindergarten site, and (c) a cluster-level paired *t* test comparing covariate-adjusted classroom means within each kindergarten. Results are summarized in Table 7.

**Table 7:** Sensitivity analyses accounting for clustering.

Analysis	Model / Key Predictor	*B*	*SE*	*p*	95% CI	Marginal *R*²	Conditional *R*²
RQ1	OLS + site fixed effects (primary)	3.75	0.72	< .001	[2.32, 5.18]	—	—
RQ1	HLM: kindergarten random intercept	3.75	0.71	< .001	[2.36, 5.15]	.71	.77
RQ1	HLM: classroom nested within kindergarten	3.74	1.24	.030	[1.31, 6.18]	.71	.78
RQ1	Cluster-level paired *t* test (5 pairs)	3.75	—	.046	[0.10, 7.40]	—	—
RQ2	OLS + site fixed effects (primary)	0.14	0.05	.004	[0.04, 0.23]	—	—
RQ2	HLM: kindergarten random intercept	0.14	0.05	.003	[0.05, 0.23]	.72	.79
RQ2	HLM: classroom nested within kindergarten	0.14	0.05	.003	[0.05, 0.23]	.72	.79

*Note.* RQ1 models: *N* = 250; posttest CT regressed on centered pretest CT, condition, age, gender, father’s and mother’s education, household income, and home language. RQ2 models: *n* = 125 (intervention group only); same covariates plus centered app engagement index. The cluster-level paired *t* test compared covariate-adjusted classroom residual means within each kindergarten site. HLM = hierarchical linear model.

For RQ1, the intervention effect remained statistically significant across all sensitivity specifications. The mixed-effects model with kindergarten random intercepts yielded an estimate nearly identical to the primary OLS model (*B* = 3.75, *SE* = 0.71, *p* < .001; marginal *R*² = .71, conditional *R*² = .77). Critically, the mixed-effects model with classroom nested within kindergarten—which directly accounts for the unit of randomization—also confirmed the intervention effect (*B* = 3.74, *SE* = 1.24, *p* = .030; marginal *R*² = .71, conditional *R*² = .78). As expected, the standard error increased from 0.72 (OLS) to 1.24 when classroom-level clustering was modeled, reflecting the reduced effective degrees of freedom with only ten classrooms. The classroom-level variance component was estimated at 2.65 (*SD* = 1.63), and the model did not exhibit singular fit, indicating that meaningful classroom-level variation existed after accounting for kindergarten-level differences. A complementary cluster-level analysis, in which covariate-adjusted residual means were computed for each classroom and compared via paired *t* test within each kindergarten site (5 pairs), corroborated the direction and significance of the effect (mean difference = 3.75, *t*(4) = 2.85, *p* = .046).

For RQ2, the engagement effect within the intervention group similarly remained significant when kindergarten random intercepts were modeled (*B* = 0.14, *SE* = 0.05, *p* = .003; marginal *R*² = .72, conditional *R*² = .79). The classroom-nested model yielded a comparable estimate (*B* = 0.14, *SE* = 0.05, *p* = .003; marginal *R*² = .72, conditional *R*² = .79), although the smaller number of intervention-group classrooms (*k* = 5) limits the precision of classroom-level variance estimates in this subsample.

The consistency of point estimates across all analytic approaches (Table 7) strengthens confidence that the primary findings are robust to how the clustered structure is modeled, including analyses that explicitly account for the classroom as the unit of randomization. We note, however, that the wider confidence intervals observed in the classroom-nested models reflect the inherent imprecision of designs with a small number of clusters.

## Discussion

This cluster-randomized trial examined the effectiveness of a 12-week interactive STEM app intervention on computational thinking skills among young children (ages 4–6) and explored whether app engagement predicted learning outcomes. Results provided strong empirical support for both primary hypotheses, demonstrating that (a) the intervention produced significant improvements in computational thinking compared to traditional instruction (Cohen’s *d* = 0.39), and (b) within the intervention group, children exhibiting higher levels of app engagement achieved superior posttest performance, accounting for unique variance beyond baseline ability (Δ*R*² = .015)—a statistically significant but practically modest association specific to the intervention context. These findings make important contributions to early childhood education research and practice.

### Main intervention effect: Comparison with prior research

Children who participated in the interactive STEM app intervention demonstrated computational thinking scores that were, on average, 3.75 points higher than controls after adjusting for baseline ability and demographic covariates—a statistically significant effect (*p* < .001) corresponding to a small-to-moderate Cohen’s *d* of 0.39. This effect size aligns closely with findings from prior computational thinking interventions in early childhood, though our study’s extended duration and larger sample provide more robust evidence.

Louka and Papadakis (2024) reported varied effect sizes across different computational thinking dimensions in their three-week ScratchJr intervention with 34 preschoolers [1]. While they documented significant improvements in representation, algorithms, and hardware-software interaction understanding, their intervention duration was only one-quarter the length of ours. Our sustained 12-week implementation suggests that computational thinking benefits accumulate over time rather than plateauing quickly, supporting the value of extended systematic instruction. Moreover, Hachey (2013) noted that their control group initially outperformed the experimental group at pretest, whereas our careful matching procedures achieved baseline equivalence, strengthening causal inferences about intervention effects [16].

Caballero-González and Muñoz-Repiso (2019) similarly documented significant pretest-posttest improvements in computational thinking among 44 preschool children exposed to programming challenges through tangible and graphical interfaces [3]. However, their study lacked a control group, limiting ability to isolate intervention effects from general developmental progression or practice effects. Our controlled design demonstrates that observed gains genuinely reflect intervention impact rather than maturation or repeated testing. Additionally, several prior studies employed researcher-developed interfaces requiring specialized equipment [17–19], whereas our use of educational tablet apps deployable on standard classroom devices enhances practical feasibility and scalability for typical early childhood settings.

The physical-digital integration approach investigated by Esther-del-Moral-Pérez et al. (2025) with 82 children aged 4–6 yielded promising results through game-based storytelling with robot characters [2]. Their pre-experimental design documented substantial computational thinking activation through observational assessment. While their approach emphasizes embodied, narrative-rich learning potentially supporting deeper engagement, the lack of standardized outcome measures and control conditions limits comparisons with our findings. Our use of the validated TechCheck-K instrument provides standardized evidence of learning gains, complementing the rich qualitative insights from observational studies [2,20–22].

Yu et al.’s (2020) CodeAttach system engaging preschoolers in computational thinking through physical play activities represents an innovative design approach [4]. However, their design-focused research emphasized feasibility and usability rather than learning outcomes. Our study complements such design research by providing rigorous effectiveness evidence, demonstrating that thoughtfully designed technological interventions—whether physical-digital hybrids or pure app-based approaches—can successfully enhance young children’s computational thinking when implemented systematically.

### Mechanisms underlying intervention effectiveness

Several interconnected mechanisms likely explain our intervention’s effectiveness, grounded in developmental and learning theories. First, the interactive apps provided immediate, individualized feedback enabling children to rapidly iterate on problem-solving attempts [18,23,24]. This aligns with formative assessment principles emphasizing that timely, specific feedback supports learning by helping learners identify errors, test revised strategies, and construct accurate mental models [25–27]. Young children particularly benefit from immediate feedback given their developing metacognitive monitoring capacities and limited ability to detect errors through pure reflection [28].

Second, gamification elements embedded within the apps—progressive challenges [29], visual rewards [30], engaging narratives [31]—likely sustained motivation across the 12-week intervention. Kim et al. (2019) demonstrated that structured creative problem-solving programs significantly enhanced both creativity and character development among 42 five-year-old Korean children, suggesting that well-designed curricula maintaining engagement can produce multifaceted developmental benefits [32]. Similarly, Pila et al.’s (2019) evaluation of Daisy the Dinosaur and Kodable with 28 preschoolers revealed that app design features—particularly scaffolding quality, feedback systems, and challenge progression—critically influence learning effectiveness [7]. Our intervention’s success likely reflects similar design principles, with the apps’ game-like features supporting sustained practice necessary for computational thinking skill consolidation.

Third, touchscreen interfaces offered intuitive [33], low-barrier interaction modalities reducing cognitive load associated with device operation [28,34,35]. This enabled children to focus cognitive resources on computational thinking concepts rather than interface mechanics. The direct manipulation affordances of touchscreens may facilitate young children’s learning by creating tighter coupling between actions and outcomes, supporting development of cause-effect understanding central to computational thinking [13,36].

Fourth, the structured 12-week curriculum created intentional developmental progressions from simple to complex tasks, scaffolding increasingly sophisticated computational thinking competencies. Rather than isolated technology exposure, the systematic progression likely enabled cumulative learning where earlier skills provided foundations for later challenges. This aligns with learning trajectory frameworks emphasizing that effective instruction sequences activities according to empirically-documented developmental progressions rather than arbitrary orderings.

### Engagement as a predictor of learning outcomes: Theoretical and practical implications

Beyond demonstrating overall intervention effectiveness, this study uniquely examined how children’s engagement patterns related to computational thinking gains—an analysis rarely conducted in early childhood technology research. Results revealed that app engagement significantly predicted posttest performance after controlling for baseline ability (*B* = 0.14, *p* = .004), with the composite engagement index accounting for approximately 1.5% of unique variance (Δ*R*² = .015, Cohen’s *f*² = 0.075).

This finding aligns with broader educational research emphasizing engagement’s predictive role in learning [4,37]. While effect size was modest, this represents meaningful prediction given stringent controls for baseline computational thinking—arguably the strongest predictor of posttest performance.

At the same time, the practical magnitude of the engagement effect warrants careful interpretation. The 1.5% of unique variance explained corresponds to a predicted posttest difference of approximately 1.3 points (on the 0–100 scale) per standard-deviation difference in engagement—roughly 0.13 *SD* of the posttest distribution. Statistical significance should therefore not be equated with educational significance: engagement is best understood as one contributor among several, embedded in a model dominated by baseline ability. Moreover, because engagement was assessed only within the intervention group, the association cannot be generalized beyond children participating in structured app-based interventions of this kind, and the direction of causality cannot be established—children with stronger emerging computational thinking skills may also have engaged more deeply with the apps. We therefore refrain from claiming a universal or causal role for engagement; rather, our findings indicate that, within this intervention context, children with higher observed engagement tended to have higher posttest scores within the intervention context. The multidimensional engagement index captured quality indicators (task completion rates, persistence, self-correction, strategic help-seeking, navigation independence) beyond simple quantity metrics (time on task, attendance). This distinction proved critical: children who not only attended sessions but also engaged deeply—completing challenging tasks, persisting through difficulties, independently correcting errors—tended to achieve higher posttest computational thinking scores.

These patterns suggest the apps successfully elicited cognitive engagement (mental effort, strategy use, metacognitive monitoring) rather than merely superficial behavioral participation. Pila et al. (2019) noted that app design features influence both engagement quality and learning outcomes, with more structured apps (Kodable) producing superior conceptual understanding compared to open-ended exploration apps (Daisy the Dinosaur) [7]. Our findings extend this work by quantifying specific engagement dimensions predicting learning, providing empirical guidance for design priorities.

The engagement variability observed within our intervention group (*M* = 72.45, *SD* = 12.38 on 0–100 scale) indicates that identical app access produced substantially different usage patterns. This heterogeneity raises important questions about individual differences and contextual factors influencing engagement. Peleg and Levy (2025) documented significant gender differences in makerspace interventions, with boys showing greater creativity gains while both genders experienced declines in tool-use self-efficacy, particularly pronounced among girls [8]. Such findings underscore that technology-enhanced learning environments may differentially impact subgroups, necessitating attention to equity and inclusive design.

### Implementation Quality and Teacher Roles

Although apps provided automated instruction and feedback, teachers played crucial facilitation roles in our intervention through training implementation, ongoing encouragement, logistical support, and integration with classroom activities. Research suggests teacher facilitation significantly amplifies educational technology effectiveness, yet our study did not systematically assess implementation quality or teacher-child interactions during app use [38–40].

Gaias et al. (2022) evaluated a math and science storytime program at public libraries, documenting significant improvements in caregiver involvement and children’s STEM engagement [41]. Their study underscores that adult facilitation and encouragement critically shape children’s learning experiences. While their context (informal library programs) differs from our formal classroom intervention, the principle that adult support enhances technology-based learning likely generalizes. Future research should examine how varying levels of teacher scaffolding, questioning strategies, and integration with non-digital activities influence both engagement and learning outcomes.

The structured implementation guides and weekly learning objectives provided to teachers in our intervention likely contributed to consistent effectiveness across sites. However, natural variation in teacher enthusiasm, technological proficiency, pedagogical skill, and adherence to protocols may have influenced outcomes [40,42,43]. Some classrooms may have implemented the intervention with high fidelity—teachers actively facilitating, making connections to other learning, encouraging persistence—while others provided minimal support beyond basic logistics. Understanding this implementation variability and its effects on outcomes represents an important direction for future research, potentially identifying optimal facilitation practices to maximize intervention effectiveness.

### Developmental Appropriateness and Screen Time Debates

Our findings contribute to ongoing debates regarding developmentally appropriate technology use in early childhood. The intervention involved approximately 60 minutes weekly across three 20-minute sessions—falling within recommended screen time guidelines when balanced with other activities. Results suggest that carefully designed, educationally purposeful technology use can produce meaningful cognitive benefits supporting computational thinking development.

However, comprehensive evaluation requires examining potential effects beyond computational thinking. Peleg and Levy (2025) documented that while makerspaces enhanced boys’ creativity, both genders experienced self-efficacy declines regarding tool use—an unintended negative outcome [8]. Similarly, technology interventions might inadvertently affect social-emotional development, physical activity levels, attention capacities, or dispositions toward learning. Our study assessed only computational thinking outcomes; future research should employ comprehensive assessment batteries examining cognitive, social-emotional, physical, and motivational domains to provide balanced evidence regarding technology integration effects.

Edwards (2017) argues that the critical question is not whether to use technology but how to integrate it appropriately within play-based, relationship-rich early childhood pedagogy [6]. Our structured intervention embedded app use within regular classroom routines with teacher facilitation and peer interaction opportunities, exemplifying thoughtful integration. However, we did not experimentally compare integrated versus isolated technology use, leaving unclear whether embedding apps within broader pedagogical contexts amplified effects. Future research might compare children using apps independently versus with adult co-engagement versus in collaborative peer groups to identify optimal social configurations for app-based learning.

Additionally, comprehensive evaluation of technology integration requires examining potential effects beyond computational thinking. Incorporating transfer tasks, naturalistic observations, teacher ratings, and open-ended challenges would provide stronger evidence regarding the depth and breadth of learning outcomes, as discussed in the Limitations section.

### Comparison with Other App-Based and Technology-Enhanced Interventions

To situate the present findings within the broader landscape of technology-enhanced computational thinking interventions for young children, it is informative to compare our results with those reported for other available educational platforms and tools. Our intervention, which used educational tablet apps over 12 weeks, produced a small-to-moderate effect (Cohen’s *d* = 0.39) on computational thinking outcomes as measured by the TechCheck-K. This effect size is broadly consistent with the range reported in a recent meta-analysis by Simonsmeier et al. (2025), who synthesized evidence across programming interventions in early childhood and reported pooled effects in the small-to-moderate range, with considerable variability depending on intervention modality, duration, and assessment approach [10].

Among studies examining specific app-based platforms, Pila et al. (2019) evaluated Daisy the Dinosaur and Kodable—two freely available coding apps—with 28 preschoolers in a pre-post design [7]. They found that Kodable, which provided a more structured progression of challenges, yielded superior gains in coding concepts relative to Daisy the Dinosaur, which offered more open-ended exploration. Our findings are consistent with this pattern, as our intervention likewise employed structured app activities with progressive difficulty aligned to Bloom’s taxonomy. Notably, our study extends Pila et al.’s work through the inclusion of a control group and a substantially larger sample, providing stronger causal evidence for the effectiveness of structured app-based approaches.

Su et al. (2024) examined the effects of cooperative play and direct instruction using an AI-enhanced educational app on kindergarteners’ computational thinking [44]. Their study reported that cooperative play conditions produced significantly greater computational thinking gains than direct instruction alone, highlighting the importance of social interaction modalities in app-based learning. Although our study did not experimentally compare instructional formats, the teacher-facilitated, classroom-embedded implementation adopted here shares features with cooperative learning approaches, and the positive effects we observed may partly reflect benefits of social learning contexts.

Comparing app-based and robotic approaches, Yang et al. (2024) directly compared tablet-based coding instruction with robot-based embodied learning among preschoolers and found that both modalities significantly improved computational thinking, though the robotic condition showed advantages in spatial reasoning and embodied cognition measures [45]. Our tablet-based intervention produced comparable computational thinking effect sizes, suggesting that well-designed app interventions can achieve outcomes similar to more resource-intensive robotic platforms, with the added advantage of lower implementation costs and greater scalability. Similarly, Del-Moral-Pérez et al. (2025) examined a multi-modal approach combining micro-stories, robotic coding, digital applications, and augmented reality for enhancing children’s computational thinking [46]. Their integrated approach yielded improvements across multiple computational thinking dimensions, and their findings converge with ours in supporting the value of structured, multi-faceted technology-enhanced interventions in early childhood settings.

In the domain of curriculum-level interventions, Bers et al. (2023) reported on the Coding as Another Language (CAL) curriculum implemented through ScratchJr across diverse school settings [11]. The CAL approach, grounded in a developmentally appropriate pedagogical framework, documented consistent improvements in children’s computational thinking and coding skills. Our research-prototype app-based approach shares with CAL the emphasis on structured curriculum design and teacher facilitation, while differing in the use of purpose-built research prototypes rather than a researcher-developed open-source platform. The convergence of positive findings across these varied approaches suggests that the quality of instructional design and implementation support may be more critical than the specific technological platform employed.

Kim et al. (2021) conducted a meta-analysis of educational app effects on preschool through grade 3 children’s learning outcomes and reported a moderate overall effect size across studies, with larger effects observed when apps incorporated adaptive features, embedded feedback, and structured learning progressions [47]. Our app engagement findings align with this pattern: the positive association between multidimensional engagement and computational thinking outcomes suggests that app features promoting active, persistent interaction—rather than passive consumption—are key drivers of learning gains.

### Design Considerations for Educational Apps in Early Childhood Computational Thinking

The findings of this study, considered alongside the broader literature, illuminate several important design principles for educational apps targeting computational thinking in early childhood. Understanding how app design features relate to learning outcomes carries practical significance for both developers and educators selecting tools for classroom use.

First, progressive challenge structures appear critical for sustaining engagement and supporting learning gains. Our intervention apps featured built-in difficulty progressions aligned with the Bloom’s taxonomy framework described in the Method section, and the positive association between engagement and learning outcomes suggests that these structures were effective. This is consistent with Callaghan and Reich’s (2021) finding that leveling designs in mobile apps—where task difficulty increases systematically as children demonstrate mastery—scaffold preschool learning more effectively than static difficulty presentations [18]. Hirsh-Pasek et al. (2015) similarly argued that educationally effective apps should incorporate “guided exploration” features that channel children’s interactions toward learning goals without eliminating agency or play, a principle instantiated in our structured-yet-interactive curriculum design [48].

Second, feedback quality and immediacy emerge as important design features. The apps used in our intervention provided immediate visual and auditory feedback for both correct and incorrect responses, enabling children to detect errors and self-correct—a behavioral pattern captured in our engagement composite index. Matthews et al. (2025) demonstrated that novel computational thinking tools for very young children are most effective when feedback is multimodal, socially distributed, and embedded within the play interaction rather than presented as an external evaluative signal [49]. Mallawaarachchi et al. (2023) conducted a content analysis of persuasive design features in early childhood apps and found that apps incorporating timely feedback, praise, and reward mechanisms showed greater user engagement, while apps relying on extraneous entertainment features (e.g., unrelated animations, distracting sounds) showed lower educational effectiveness [50]. These findings reinforce the importance of feedback systems that are integral to the learning task rather than tangential to it.

Third, minimizing extraneous cognitive load through intuitive interface design is particularly important for young children. Clarke-Midura et al. (2019) compared the design features of early childhood coding toys and found that tools requiring fewer symbolic abstractions and perspective-taking demands were more accessible to the youngest users [51]. Our use of touchscreen-based apps with direct manipulation interfaces likely reduced the interface-related cognitive demands that can impede learning with more complex robotic platforms, allowing children to allocate cognitive resources toward computational thinking concepts rather than device operation.

Fourth, the capacity for social and collaborative interaction warrants attention in app design. Although our apps were designed for individual use on tablets, the classroom-embedded implementation created natural opportunities for peer observation, discussion, and collaborative problem-solving facilitated by teachers. Lennon et al. (2022) found that children’s social and independent behaviors during play with a coding app varied significantly based on digital game features, with certain design elements promoting more collaborative interaction than others [52]. Future app designs for early childhood computational thinking should explicitly incorporate features that support joint engagement between children and between children and adults, building on the distributed interaction design principles described by Matthews et al. (2025) for tangible computational thinking tools [49].

Finally, the alignment between app activities and curricular learning objectives is essential. Griffith et al. (2020) noted in their systematic review that apps producing the largest learning gains were those whose activities were tightly aligned with specific educational goals and integrated into structured instructional contexts, rather than used as standalone entertainment or free-play resources [53]. Our study operationalized this principle through the Bloom’s taxonomy-aligned curriculum framework, weekly learning objectives, and teacher facilitation guides, which together ensured that app interactions served targeted educational purposes.

### Practical implications

Despite limitations, findings offer actionable implications for practice. First, results support integrating high-quality interactive STEM apps into early childhood curricula as effective computational thinking tools. The moderate effect size and robust findings across sites suggest apps represent valuable instructional resources when thoughtfully selected and systematically implemented.

Second, although the unique variance explained by engagement was modest in the present study, implementation should prioritize fostering deep engagement through: (a) modeling effective navigation and problem-solving, (b) encouraging verbalization of thinking, (c) celebrating persistence and error correction, (d) facilitating peer collaboration, and (e) connecting app activities to computational thinking concepts. Professional development supporting these facilitation practices would likely amplify effects.

Third, app selection should prioritize educational quality over entertainment appeal. Apps minimizing distracting features while maximizing educational substance tend to produce superior learning [7]. Teachers need support identifying high-quality options through professional development, curated libraries, and evaluative tools.

### Limitations and future research directions

Despite methodological strengths, several limitations warrant acknowledgment. First, although 12 weeks represented substantial extension beyond typical brief interventions [1,3], longer-term investigations with delayed posttests are needed to assess whether gains persist, fade, or compound over time. Follow-up assessments at 6-month and 12-month intervals would clarify durability and inform decisions about ongoing support needs.

Second, although classrooms were randomly assigned to conditions within each kindergarten, the number of randomized clusters was modest (ten classrooms), and unmeasured classroom-level confounds (classroom climate, teacher characteristics, peer dynamics) may have influenced outcomes. The cluster-based power analysis revealed that the design provided approximately 49% power to detect the target effect size of *d* = 0.40, which is below the conventional 80% threshold. This underpowering is inherent to the small number of clusters rather than the individual-level sample size, and it is reflected in the wider confidence intervals observed in the classroom-nested sensitivity analyses. The consistency of results across the primary analysis and all three sensitivity specifications (kindergarten random intercepts, classroom-nested random intercepts, and cluster-level paired *t* test) strengthens confidence in the findings; nevertheless, future trials with a substantially larger number of randomized clusters would provide more precise and generalizable estimates.

Third, the five participating kindergartens were selected through convenience sampling based on established research collaboration agreements rather than through probability sampling from the broader population of kindergartens in Northern China. Although the sites varied in size, socioeconomic catchment area, and curricular emphasis, the findings may not generalize to all kindergarten settings in the region, particularly those with substantially different resource levels, teacher qualifications, or student demographics.

Fourth, the study did not implement a formal protocol for managing missed intervention sessions. While implementation fidelity monitoring confirmed that at least 80% of planned sessions were completed across classrooms, individual children may have missed sessions due to illness, family circumstances, or other reasons. The absence of a minimum attendance threshold for inclusion in the analytic sample means that some children may have received lower dosages of the intervention than others, potentially attenuating the observed effects. Future research should establish explicit attendance criteria and examine dose-response relationships between the number of sessions completed and learning outcomes.

Fifth, computational thinking assessment relied exclusively on TechCheck-K. Although this instrument has demonstrated strong psychometric properties and sensitivity to interventions, incorporating transfer tasks, naturalistic observations, teacher ratings, and open-ended challenges would provide stronger evidence regarding depth and breadth of learning [19].

Sixth, because the three educational apps were research prototypes developed for the present study, independent replication requires access to these materials. Representative screenshots, task examples, activity protocols, scoring/interaction rules, and relevant technical specifications are available from the corresponding author upon reasonable request.

Seventh, app usage engagement was measured only within the intervention group, because the construct is defined by children’s interaction with the intervention apps. The observed association between engagement and computational thinking outcomes is therefore predictive rather than causal, is specific to the intervention context, and explained a modest proportion of unique variance (1.5%); it should not be generalized to young learners more broadly. Future research that experimentally varies conditions expected to influence engagement (e.g., dosage, facilitation intensity, app features) would permit stronger inferences about the causal role of engagement in technology-based learning.

## Conclusion

This study demonstrates that a structured, 12-week interactive STEM app program can meaningfully improve computational thinking skills in young children when embedded within regular kindergarten classroom routines with teacher support. The intervention produced a small-to-moderate improvement in computational thinking relative to traditional instruction, indicating that educational tablet apps represent a viable and scalable tool for early childhood computational thinking education. Importantly, the gains observed were not attributable to baseline differences between groups, demographic factors, or site-level variation, strengthening confidence that the intervention itself drove the observed improvements.

Beyond the overall effectiveness of the intervention, this study reveals that, within the context of a structured app-based intervention, how children engage with educational apps is associated with what they learn. Children who interacted more deeply with the apps—completing challenging tasks, persisting through difficulties, self-correcting errors, and navigating independently—achieved somewhat stronger computational thinking outcomes than children who participated more passively, although the unique contribution of engagement was modest and its generalizability beyond the intervention context remains to be established. This finding suggests that merely providing access to educational technology may be insufficient; fostering active, sustained, and cognitively effortful engagement is likely important for maximizing learning benefits—a hypothesis that future experimental work should test directly. For educators, this underscores the importance of teacher facilitation strategies, thoughtful app selection, and structured curricular integration in translating technology access into meaningful learning.

These findings contribute to the growing evidence base supporting constructivist approaches to technology-mediated learning in early childhood [6]. The results suggest that when educational apps are carefully selected, aligned with developmental learning progressions, and implemented within supportive instructional contexts, they can serve as effective platforms for building foundational cognitive competencies. At the same time, the design of both the apps and the surrounding instructional context appears to be critical; the convergence of our findings with those from studies using varied technological platforms [11,44,46] points to the quality of instructional design and implementation—rather than the technology itself—as the primary driver of learning outcomes.

Looking ahead, several important questions remain. Whether the computational thinking gains observed here persist over months or years after the intervention concludes is unknown and warrants longitudinal follow-up. The mechanisms through which individual differences in engagement arise—and whether specific subgroups of children benefit more or less from app-based instruction—deserve further investigation [8]. Research comparing app-based, robotic, and unplugged approaches within a single experimental framework would clarify the relative strengths and limitations of each modality. Finally, efforts to promote equitable access to high-quality educational technology, particularly in under-resourced early childhood settings, are essential for ensuring that the benefits of computational thinking education reach all children [32]. By addressing these questions, future work can help realize the promise of technology-enhanced learning while safeguarding against potential risks, ultimately supporting young children’s preparation for participation in an increasingly computational world.
